# Experimental and Numerical Analysis of Thermal Fatigue of Grey Cast Iron Ingot Mould

**DOI:** 10.3390/ma17235735

**Published:** 2024-11-23

**Authors:** Piotr Mirek, Jarosław Piekło, Aldona Garbacz-Klempka

**Affiliations:** 1 Krakodlew S.A., Ujastek 1 St., 30-969 Kraków, Poland; pmirek@agh.edu.pl; 2 Faculty of Foundry Engineering, AGH University of Krakow, Reymonta 23 St., 30-059 Krakow, Poland; jarekp60@agh.edu.pl

**Keywords:** grey cast iron, thermal fatigue, ingot mould, temperature analysis, strength analysis, microstructural analysis

## Abstract

This article presents the results of experimental studies and numerical calculations that were conducted to analyse the phenomena that occur during the operation of an ingot mould that is designed for casting steel ingots. The studies were conducted on an experimental stand in a foundry on an ingot mould that was designed to make ingots that weigh up to six tons; they consisted of determining the temperature of the ingot mould and measuring the displacements of its walls during filling with steel and cooling. These studies were used to create and verify a numerical model that was used to determine the temperatures, displacements, deformations, and stresses in ingot mould walls during the operating cycle using the FEM method. Microstructure studies of ingot cast iron that was subjected to thermal fatigue were also conducted on a laboratory stand; the temperature changes and test times were the same as those used under the normal operating conditions of the ingot mould. Cast iron samples were subjected to heating and cooling cycles within a range of 0 to 60 cycles; then, tensile tests were performed to determine their stress–strain curves. As a result of the conducted tests, a great influence was found of the number of cycles on decreases in the values of the modulus of elasticity and tensile strength—especially within a range of 0 to 10 cycles. A relationship was also found between the changes in these values and the image of the cast iron microstructure. Based on images of the cast iron microstructure after being subjected to different numbers of thermal fatigue cycles, the mechanism of the crack initiation and propagation was determined. The influence of the changes in the strength of the cast iron and the stress state that was determined by the FEM method on the durability of the tested type of ingot mould was analysed. The obtained research results will be useful for introducing design changes that are aimed at increasing the fatigue durability of ingot moulds.

## 1. Introduction

The phenomenon of fractures in metals that result from the occurrences of cyclic thermal stresses that are induced by heating or cooling is usually referred to as thermal fatigue or thermal shock (when the rate of the heating or cooling is rapid [1,2,3]). Components that are subjected to thermal fatigue include engine heads, exhaust manifolds, and brake discs in the automotive industry, dies and the stamping of dies in metal forming, and ingot moulds in the steel industry [4].

Ingots and the cast iron moulds that are used for casting steel ingots are typical examples of castings that are subjected to thermal fatigue during their operation. The weight of these castings ranges from several tons to more than a hundred tons, and the wall thicknesses can be as much as 400 mm. While filling its interior with steel, the temperature of the inner wall of an ingot reaches temperatures that are within a range of 750 °C to 1000 °C, and the temperature gradient can exceed 4 K/mm in some parts of the ingot. After the ingot is removed from the mould, it cools down to the ambient temperature; in some cases, this is accelerated by the action of a water mist or water jet (after which, the ingot is again filled with steel). Cyclic temperature changes (heating–cooling) of the walls of the ingot cause changes in the structure of the cast iron combined with changes in the volumes of some parts of the ingot; these changes are the cause of the appearance of a network of cracks on the surfaces of the walls that are in contact with the metal. In turn, the large temperature gradient that occurs between the inner and outer walls of the ingot is the cause of large deformations and stresses; their consequence is the propagation of surface cracks and, consequently, the formation of cracks passing through the wall of the ingot casting [5,6]. Cracks of this type lead to the withdrawal of the ingot from further operation. The causes of these cracks include both structural and microstructural factors. The above-described phenomena result in the requirement for the ingot material to withstand the thermal fatigue, stress formation, and surface and internal oxidation that usually occur at the interface between the graphite and matrix separations. Practice and economic considerations mean that the most commonly used material for making ingot moulds is grey cast iron with flake graphite or blast furnace pig iron [7,8,9]. There is a trend to expand this range to include cast iron with ball graphite and vermicular graphite. Nevertheless, the chemical composition and alloy modification is prepared so that the index of thermal fatigue resistance is as high as possible [10,11,12].

Since there is no standard method for testing thermal fatigue cracking, a number of experimental studies have been conducted to determine the resistance to thermal load-induced cracking. In publication [13], the authors used the cyclic induction heating of a bar, air cooling it from 100 °C to 540 °C to determine the fracture properties of cast iron. In contrast, publication [14] used thin plates that were subjected to induction heating, followed by water cooling within a range of 20–800 °C. In publication [4], the authors made a casting die from ingot material, then proceeded with cyclic heat loading tests by flooding the die with liquid steel at 1450 °C. In the first stage, they used air cooling until a skin was formed; after this, they used a water mist cooling medium to intensify the performance. In the final stage, they cooled the empty die with air after the ingot was knocked out. Immediately after the cycle was completed, the test was repeated.

A number of comparative studies have also been conducted on the thermal fatigue resistance of different cast iron grades. In publication [15], the author concluded that grey cast iron had a higher resistance to thermal fatigue when compared to vermicular cast iron; this was due to its lower Young’s modulus value and its higher temperature compensation coefficient. The authors of publications [16,17,18] came to the same conclusions. In contrast, the authors in publications [4,19,20] found that vermicular cast iron had a better resistance to thermal fatigue, resulting from its higher strength-to-thermal-stress ratio.

In publication [21], the authors conducted tests on grey cast iron that was subjected to cyclic thermal fatigue by rapid heating it to 800 °C then cooling it to 20 °C. The assumed cycle time was 160 min. Samples that were subjected to cyclic heat treatment within a range of 1–50 cycles were subjected to static tensile tests within a range of 20–800 °C. A decrease in the strength of the cast iron could be observed; this was due to thermal fatigue.

The high dependence of the tensile strength and thermal fatigue resistance of grey cast iron on creep resistance at high temperatures was demonstrated in publications [22,23]. On the basis of the experiments that were conducted in publication [24], the author concluded that the reason for the formation of thermal fatigue was the reduction in the contraction and expansion of the material under cyclic thermal loads. In [25], fatigue tests (including grey cast iron) were analysed; it was discovered that, when determining the rate of the crack propagation, the plastic processing of the material played a major role.

Thermal fatigue tests were conducted on disc-shaped specimens with fins placed at one end in order to simulate brake discs [26]. As a result, it was found that the graphite morphology had the greatest influence on the fracturing of perlitic grey cast iron.

Thermal fatigue tests on an automatic line of disc-shaped specimens were carried out in publications [27,28]. Increased resistance to thermal fatigue was shown in samples with molybdenum, chromium, and copper; this also had an effect on increasing thermal conductivity. In further studies [29,30], a mathematical model for fatigue-life prediction was developed for grey cast iron.

Much attention was paid to the effects of heat treatment, alloying additives, microstructure optimisation, and casting control on thermal fatigue resistance in publications [31,32,33,34,35,36,37].

In this article, the authors focus on conducting experimental tests on the pouring station of an actual ingot to determine the changes in the temperature fields and the displacements of the ingot walls during the operating cycle. The above studies were the basis for conducting a numerical analysis using FEM software to determine the temperature, displacement, and stress fields in the walls of an ingot and the subsequent verification of the correctness of the calculations. Based on the results that were obtained through experimental tests on an actual steel ingot bench, as well as through simulations using numerical tools, cylindrical specimens were prepared for static tensile testing for which heating–cooling heat-load characteristics were developed in a high-temperature furnace. Tests were carried out on the strength properties of the ingot material that was subjected to cyclic loading. The values of the basic strength parameters of the tested material were determined, and the changes in the microstructure that were caused by cyclic thermal loading were then analysed. Based on the analysis of the changes in the microstructure, the successive stages of crack initiation and subsequent propagation of the ingot material were distinguished.

In addition to material tests for determining the effects of temperature changes on the structure and strength of the cast iron ingot, tests and studies were conducted to determine any structural assumptions for the design of the ingot castings. The results of such tests are mostly unpublished and are the property of the companies that produce such castings; however, some rules of thumb for the design and operation of ingot moulds are known. These are presented in [38]:

- Grey cast iron with flake graphite or blast furnace pig iron was considered to be the most favourable material; this was determined by the material’s economic and technological considerations, as well as its thermophysical properties.
- The average thickness of the walls of the ingot moulds was chosen to ensure the required heat capacity: the ratio of the weight of the ingot to the weight of the ingot mould was required to be between 0.7 and 1.3.
- The shape of the ingot mould needed to ensure the least-possible variation in the stress field.

Ingot moulds are usually designed on the basis of the experience that is gained during their operation. Attempts at generalisations that have been made in previous years for creating calculations and designing formulas have proven to be unsuccessful. Along with modern measuring equipment, currently available software that simulates the thermal processes and changes in the state of stress and deformation make it possible to carry out precise analyses of the phenomena that occur in this type of structure. According to the authors, it is important to distinguish between the influence of the phenomena that occur in the walls of the ingot in the zone that is adjacent to the liquid metal at a temperature of around 700 °C and the phenomena on the outer surfaces of the walls. This represents an innovative approach to the issue of thermal fatigue in ingots. In those areas where the temperature reaches temperatures that are above 700 °C, strong material degradation occurs; this can be assessed from the microstructural images and strength tests of cast iron that has been subjected to different numbers of thermal loading cycles. The outer walls are areas of crack initiation—the formation and development of which can be determined by a stress-state analysis. An analysis of the influence of the phenomena that occur in the zones that are strongly influenced by high temperatures and in the external layers of the surface of the ingot mould walls prompted the authors to carry out tests on the actual pouring station of the ingot mould in the foundry in order to precisely determine the temperature changes and displacements of the ingot mould walls; they also carried out tests on the losses of strength of the ingot mould iron under thermal fatigue. Compared to those that are usually carried out, these tests differed in terms of the characteristics of the thermal loading cycles (which were similar to those that occurred in the ingot). One cycle (lasting six hours) consisted of rapid heating and slow cooling stages. In contrast, one cycle typically lasts from several seconds to several minutes in typical thermal fatigue tests that involve grey cast iron.

## 2. Materials and Methods

The test specimens were made by casting from cast iron with the chemical composition that is shown in Table 1. The chemical composition of the specimens after casting was determined using an ARL 3460 Advantage (Thermo Scientific, Waltham, MA, United States) emission spectrometer.

**Figure 1:**
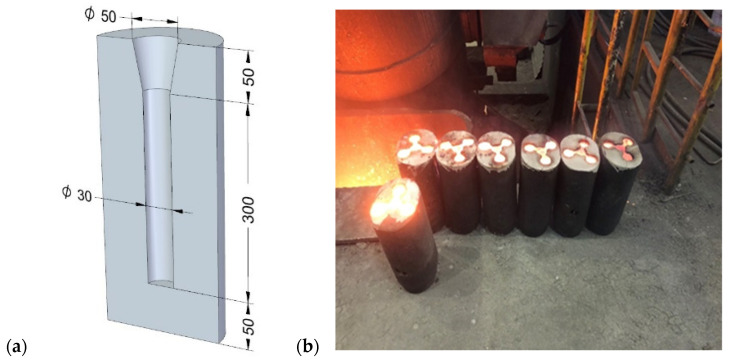
Sand moulds for separately cast trial ingots: (**a**) mould cavity dimensions; (**b**) set of moulds after pouring.

Sand moulds for the separately cast test ingots with a length of 300 mm and a diameter of 30 mm were prepared in accordance with the recommendations of EN 1561 [39]; the cast iron melting was carried out in an Otto Junker 16 Mg (Otto Junker GmbH, Jagerhausstrabe, Germany) induction furnace. Figure 1a shows the dimensions of the sand mould cavity and the set of moulds after pouring the cast iron.

**Figure 2:**
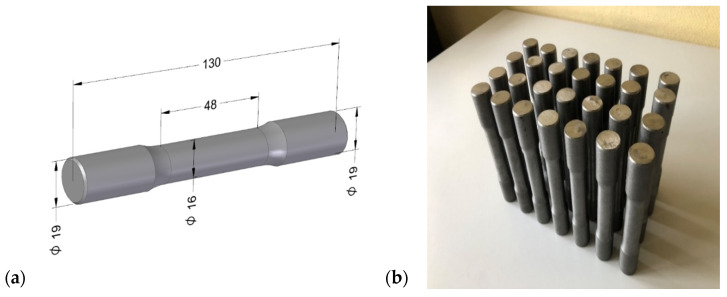
Static tensile test specimen: (**a**) specimen dimensions; (**b**) test specimen set.

Twenty-eight specimens with a gauge section diameter of 16 mm and a length of 130 mm were made from the cast ingots by machining for static tensile testing in accordance with the recommendations of PN-EN ISO 6892-1:2020-05 [40]. The specimens were made on a CNC GILDEMEISTER CTX400 (Drehmaschinen GmbH, Elsterweg, Germany) lathe. Figure 2a shows the specimens’ dimensions, and Figure 2b shows the set of finished specimens for strength testing.

The specimens were cycled before the tensile test in a Carbolite HTF 1800/4 (Carbolite Gero, Derbyshire, United Kingdom) high-temperature chamber furnace. The cycle parameters (consisting of heating, temperature stabilisation, and cooling phases) and the duration of each phase are shown in Table 2. Sets of specimens were subjected to 0 to 60 heating–cooling cycles successively (with intervals of 10 cycles).

After the heat treatment, static tensile tests were performed at room temperature in accordance with the recommendations of PN-EN ISO 6892-1:2020-05 [40]. The tests were carried out on an MTS 810 testing machine (Eden Prairie, Minnesota, USA). An MTS 634.31F-24 extensometer (Eden Prairie, Minnesota, USA) with a measuring base of 30 mm was used to measure the linear deformation. The strength tests that were carried out were designed to determine the strength loss of the cast iron as a result of the different numbers of heating–cooling cycles.

**Figure 3:**
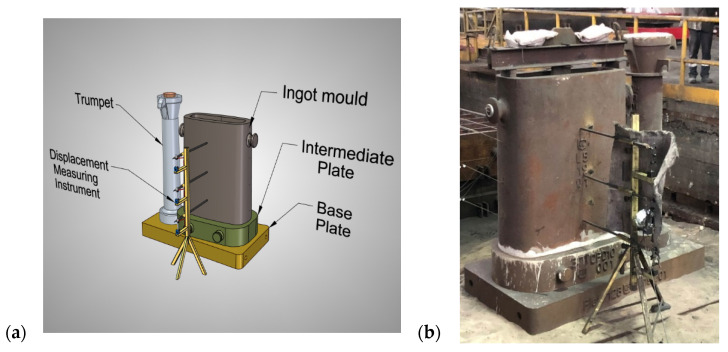
Pouring station of ingot mould: (**a**) geometrical model; (**b**) actual station.

The parameters of the thermal treatment of the samples that was carried out in the chamber furnace were determined on the basis of experimental tests that were carried out in the foundry on the actual steel ingot pouring station that is shown in Figure 3.

**Figure 4:**
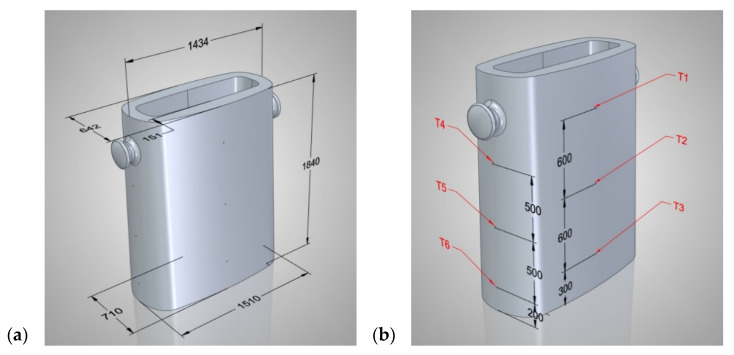
Measuring ingot: (**a**) overall dimensions; (**b**) locations of measuring points for temperature variations.

The ingot mould pouring station consisted of a 6000 kg ingot mould, a base plate, an intermediate plate, a metal feed system (siphon), and sets of sensors that measured the temperature and ingot wall deflection. The dimensions of the ingot mould are shown in Figure 4a, while the locations of Measuring Points T1, T2, T3, T4, T5, and T6 are shown in Figure 4b (along with a description). The temperature changes in the walls of the ingot box were recorded using a six-thermocouple-type PTTK-BT-60-1-SO-500 (Limatherm Sensors, Limanowa, Poland) and a FLIR T1020 (Teledyne Flir, Wilsonville, Oregon, United States) thermal-imaging camera.

**Figure 5:**
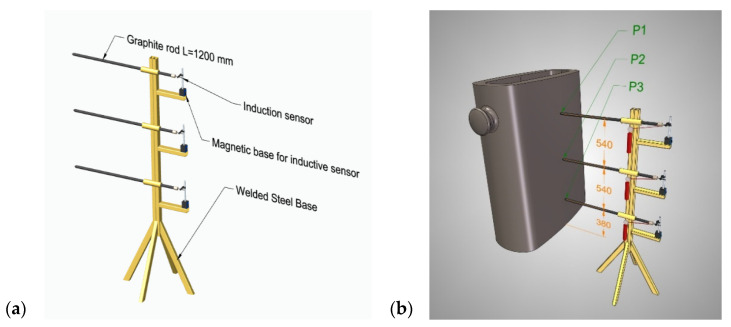
Measuring displacement of ingot: (**a**) geometrical model of device for measuring displacement of ingot walls; (**b**) arrangement of displacement measurement points.

Measurements of the displacements of the walls of the ingot during its filling with liquid melt were carried out at Points P1, P2, and P3, which are shown in Figure 5a. The displacements of the walls of the ingot were recorded using a specially designed and manufactured device (shown in Figure 5b).

The ingot displacement measuring device was a welded steel base on which graphite rods with a diameter of 20 and a length of 1200 mm were slidably mounted at three measuring heights. Due to their very low coefficient of thermal expansion (α = 6.5 × 10^−6^/°C), they formed a measuring string that limited the influence of the thermal radiation of the ingot under testing on the inductive sensors. At the end of the graphite rod, GT21-I inductive displacement sensors that were manufactured by TESA FRANCE S.A.S (Lieusaint, France) were installed on magnetic bases of a type of MDZB (L = 231 mm) with a measurement accuracy of 0.01 mm.

## 3. Numerical Model

The analyses of the temperature changes and the displacement, strain, and stress fields in the ingot model were carried out using FEM. The commercially available Siemens Simcenter NX (Version 2312/Update 8300) with the Simcenter 3D Multiphysics module (Thermal Flow software) [41,42] was used for this purpose. The software was also used to develop the characteristics of the heating of the samples inside the high-temperature furnace chamber and to determine the required furnace parameters for the different phases of the heating–cooling cycle (as shown in Table 2). In the steelmaking process of producing steel ingots in ingot moulds, there are several characteristic stages:

1. Filling the ingot mould cavity with an alloy at a specific temperature and specific pouring rate;
2. Solidification and cooling of the alloy;
3. Formation of a gap between the ingot and alloy;
4. Removal of ingot from ingot cavity and free cooling of the ingot to ambient temperature.

Initially, the surface of the mould wall is in contact with the liquid metal. After the formation of the so-called casting skin, the mould wall comes into contact with the solidified alloy. As a result of the contraction of the cooling ingot and due to the expansion of the ingot as a result of the heating, a cavity filled with hot air is later formed. Once the ingot is knocked out, the walls of the ingot cool freely to the ambient temperature. In this study, an analysis of the steel ingot manufacturing process was performed using two numerical models: thermal and stress. Due to the complexity of the process flow, a number of simplifications were required in the models that are described below.

### 3.1. Thermal Model

The durations of the individual stages that were adopted in the numerical model were the same as those used in the production cycle in the steelmaking department for the ingot structure under study. The duration of the production cycle was assumed to be 6 h (21,600 s). Table 3 shows the breakdown of the production cycle into the various stages that were adopted in the numerical model, taking their durations into account.

The geometries of the ingot mould and the steel ingot were considered in the analysed model. The geometry of the intermediate plate was neglected, and the convection boundary condition α (W/m^2^·°C) was used. A surface-to-surface contact boundary condition with the heat transfer coefficient (HTC) assignment was used to describe the heat transfer between the ingot and the mould. The free convection to ambient boundary condition α (W/m^2^·°C) was used to describe the heat transfer between the ingot and the ambient temperature. Table 4 shows the applied heat transfer boundary conditions in the analysed numerical model.

The temperature model assumed the temperature-dependent properties of the grey cast iron and the steel alloy; Table 5 overdetermines the values of the density, thermal conductivity, and specific heat that were used for the calculations. The initial ingot temperature was 20 °C, and the initial alloy temperature was 1450 °C. The numerically determined changes in the temperature field during the production cycle were taken as the heat load on the ingot material during the stress analysis in order to determine the component stress–strain tensor in its walls.

**Figure 6:**
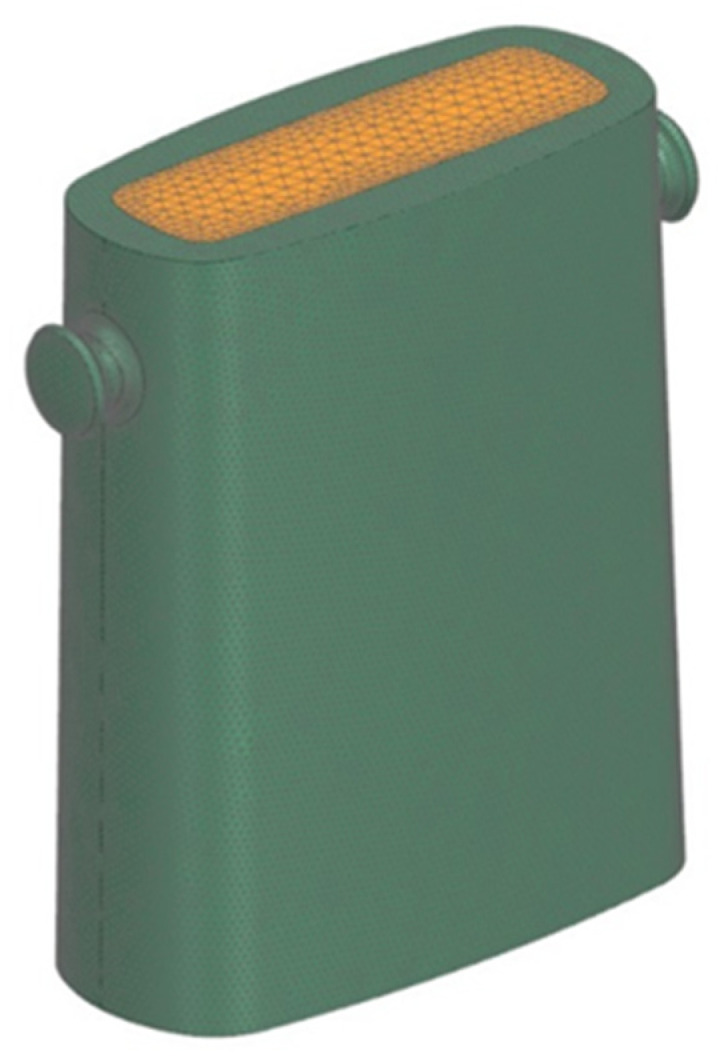
Geometrical model of ingot mould.

Figure 6 shows the geometric model of the ingot mould and the steel ingot.

### 3.2. Temperature Model of Heating–Cooling Cycle of Sample in Furnace Chamber

**Figure 7:**
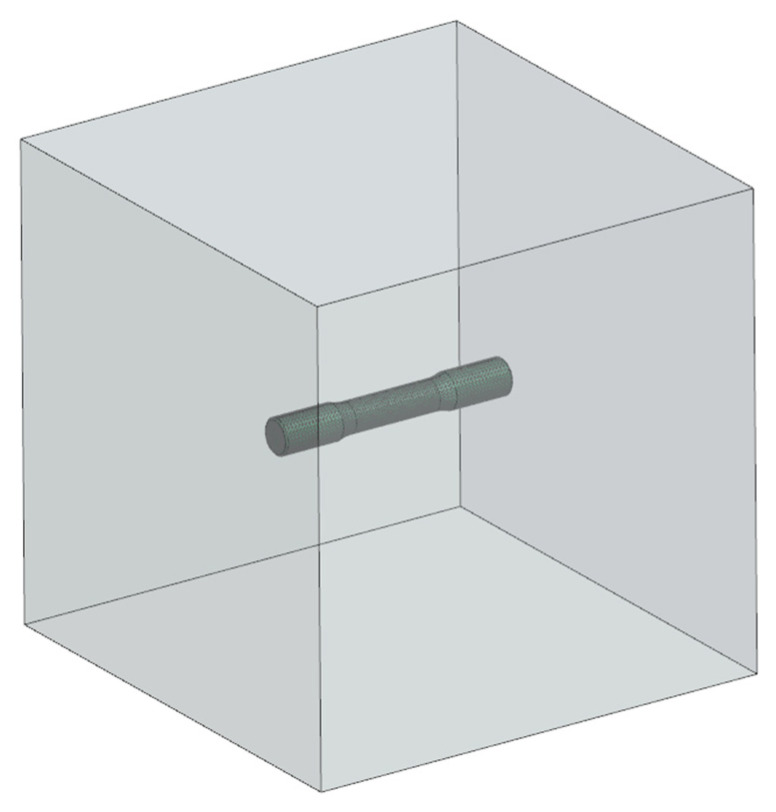
Geometrical model of furnace chamber with sample inside.

Before carrying out the cyclic thermal treatment, the heating characteristics of the prepared samples inside the high-temperature furnace chamber were developed using FEM numerical tools, and the required furnace thermal cycle parameters were determined for the individual treatment phases. For this purpose, a numerical model of the furnace chamber with the sample inside was developed. The boundary conditions for the heat transfer between the air inside the furnace chamber and the sample that is shown in Figure 7 were modelled.

In order to carry out the calculations, a model of a specimen with the dimensions that are shown in Figure 2, placed inside the chamber of a Carbolite HTF 1800/4 high-temperature furnace with dimensions of 250 × 250 × 250 mm, was adopted. The model assumed the properties of grey cast iron as a function of temperature changes (which are summarised in Table 6). Air was assumed to be the filling substance in the furnace chamber. For air, it was assumed that its properties did not change with the rising temperatures. Table 6 shows the air properties that were assumed in the numerical model.

A heat transfer coefficient (HTC) of 12 (W/(m^2^·°C) for the sample air vapour was assumed in the calculations, as was an initial sample temperature of 20 °C. The temperature of the furnace chamber was assumed according to the parameters in Table 2.

The temperature rise rate inside the furnace chamber, the holding time, and the cooling time were all determined. Based on the results of the tests that were carried out on the actual pouring station, it was assumed that a maximum temperature value of 675 °C was reached by the sample after 65 min. From this point, the sample cooled down for 6 h to a temperature of 20 °C.

### 3.3. Stress Model

Only the geometrical model of the ingot itself was included in the stress model; the other parts of the ingot stand that were present in the temperature model were removed. The stress model assumed that there were two mutually perpendicular planes of symmetry that intersected at the vertical axis of the ingot. It was assumed that the lower surface of the ingot (which was in contact with the sub-ingot plate) was not displaced in the direction of the ingot axis. The changes in thermal stress during the successive phases of the heating and cooling cycles of the ingot were determined every 300 s. The thermal load on the ingot was defined by the time-successive temperature fields that were imported from the temperature model. The finite element mesh density was the same for both the stress and temperature models. Table 7 shows the material mechanical properties that were adopted in the calculations for the stress model.

## 4. Results of Numerical Calculations

### 4.1. Heating–Cooling Cycle Parameters for Test Samples

**Figure 8:**
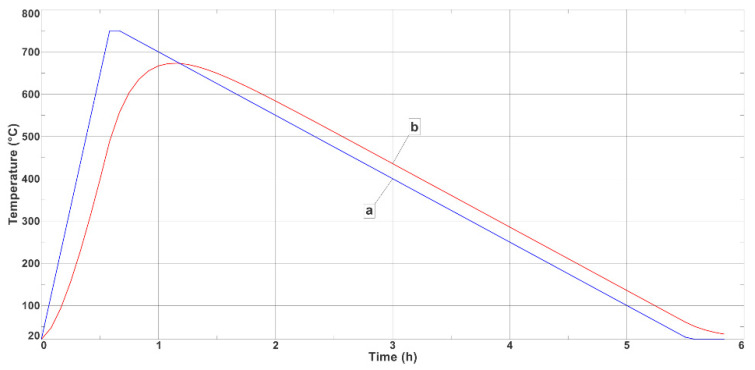
Temperature changes during thermal treatments of samples: (a) in high-temperature furnace chamber; (b) in sample (as determined by numerical FEM analysis).

Determining the high-temperature furnace setting parameters required numerical calculations that used the finite element method. The calculations were carried out using Simcenter NX software in accordance with the adopted model that was described in Section 3.2. Figure 8 shows plots of the temperature change in the furnace chamber (Curve a) during the heating–cooling cycle (the successive phases of which are given in Table 2) and the temperature change of the sample in the furnace chamber (Curve b) in which the thermal treatment was carried out.

**Figure 9:**
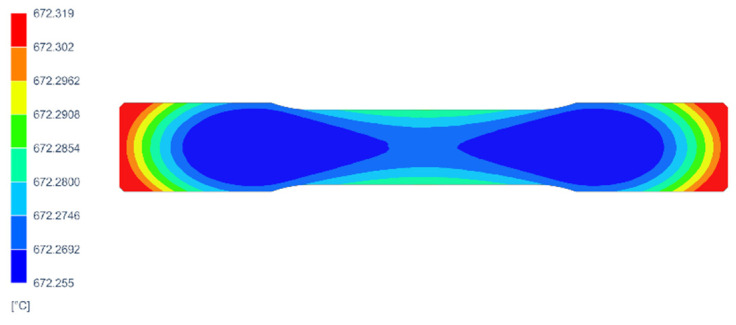
Temperature distribution on surface of sample when temperature reached its highest value.

As can be seen from the numerical calculations that were carried out, the assumed thermal parameters of the high-temperature furnace that are shown in Table 2 made it possible to achieve the required temperature changes in the specimens to be tested for durability. After 3900 s, the temperature of the specimen reached 672 °C and then decreased to room temperature in about 21,600 s. Figure 9 shows the temperature field on the surface of the specimen at the point in time that the temperature reached its highest value (672 °C).

### 4.2. Results of Ingot Temperature Field Calculations

**Figure 10:**
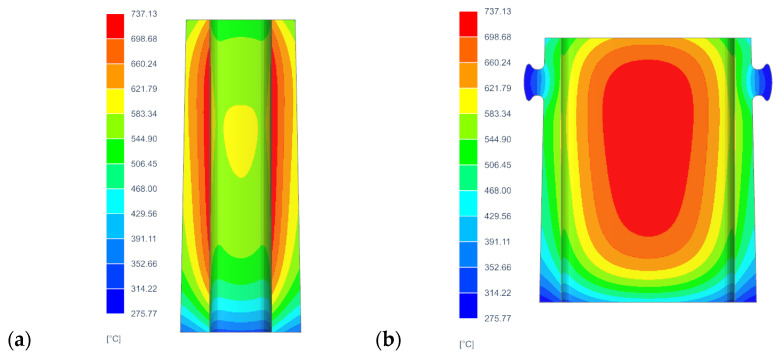
Wall cross-sections of temperature fields when ingot absorbed maximum thermal energy: (**a**) cross-section passing through centre of longer ingot walls; (**b**) cross-section passing through centre of shorter ingot walls.

The determination of the components of the stress and strain tensor inside the mould walls, as well as the development of assumptions for the thermal treatment of the samples used in the static tensile test, required the prior determination of temperature changes in the mould walls. The average temperature values of the areas of the inner and outer surfaces were taken as the reference temperatures for the development of the thermal treatment procedure for the samples in the high-temperature furnace. Figure 10a shows the temperature field via a cross-section passing through the centre of the longer walls of the mould when the temperature of the outer wall reaches the highest value. This is also the moment when the mould absorbs the maximum value of thermal energy during the production cycle. The analysis of the temperature changes in the ingot model was carried out using FEM. The calculations were carried out using Simcenter NX in the Multiphysics and Thermal Flow module. The analysis was based on the numerical temperature model described in Section 3.1. Comparisons of the temperature fields in the ingot mould wall cross-sections made it possible to determine the locations of the areas in the ingot mould walls with the highest and lowest temperatures during the production cycle. The average temperature values of the areas of the inner and outer surfaces were taken as the reference temperatures for the development of the thermal treatment procedure for the samples in the high-temperature furnace. Figure 10a shows the temperature field via a cross-section passing through the centre of the longer walls of the ingot when the temperature of the outer wall reached its highest value. This was also the moment when the ingot absorbed the maximum value of thermal energy during the production cycle. On the other hand, Figure 10b shows the temperature field via a cross-section through the centre of the shorter walls of the ingot in the same time step.

**Figure 11:**
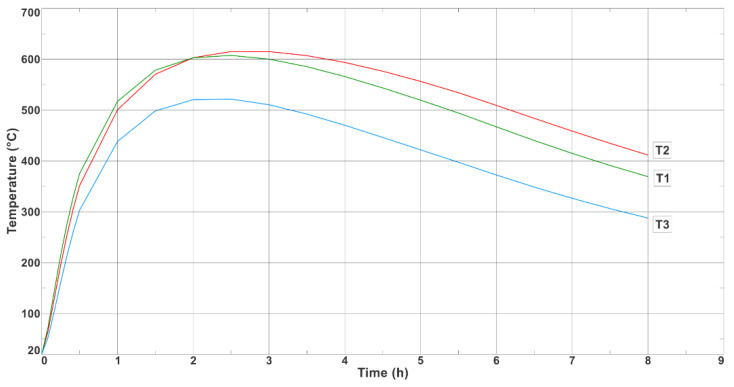
Numerically determined temperature changes at Points T1, T2, and T3 (located on long side of ingot).

**Figure 12:**
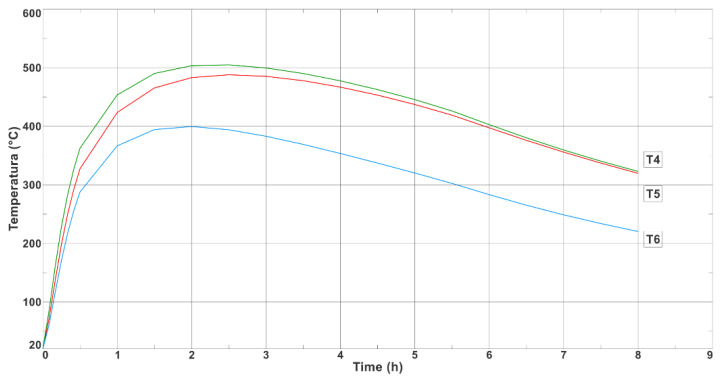
Numerically determined temperature changes at Points T4, T5, and T6 (located on long side of ingot).

Figure 11 shows the temperature-change curves on the outer surface of the longer wall of the ingot at Points T1, T2, and T3 (where the thermocouples were placed during the experimental bench tests, as shown in Figure 4b). Figure 12 shows the analogous temperature-change curves that were determined on the outer surface of the shorter ingot wall at Points T4, T5, and T6.

**Figure 13:**
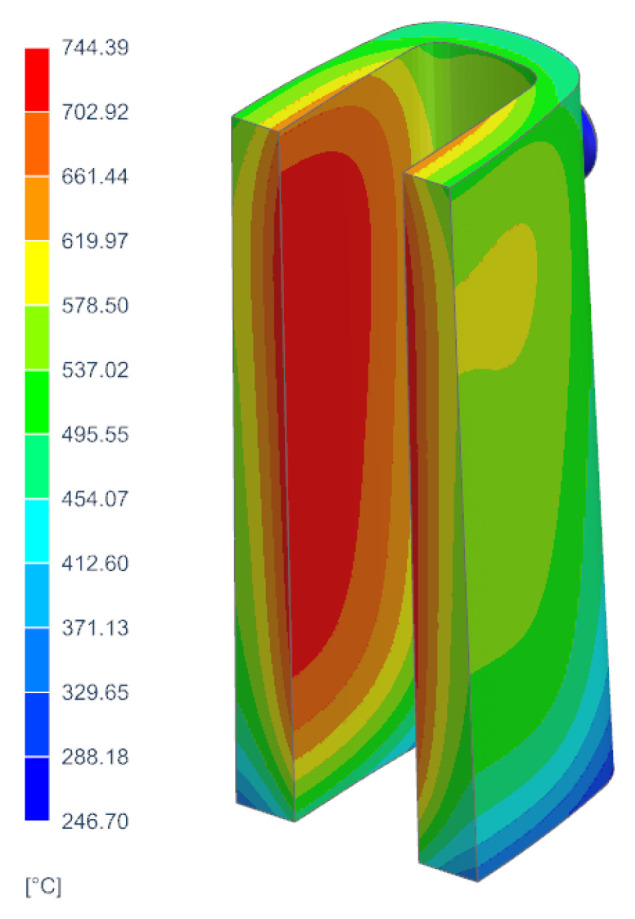
Distribution of temperature fields at maximum heating of inner wall of ingot mould.

As can be seen from the measurements, the middle and upper parts of the ingot had a temperature that was approximately 100 °C higher than in its lower part; the difference in the temperatures of these parts of the ingot during most of the production cycle can be seen in Figure 11 and Figure 12. The maximum recorded temperature of the central part of the longer side of the ingot was 622 °C (Figure 11, Curve T2), while for the shorter side, the temperature of the central part at Measurement Point T4 reached 509 °C (Figure 12). The maximum temperatures on the outer wall of the ingot appeared approximately 2 h after the ingot cavity was filled with liquid melt. Figure 13 shows the temperature field values on the inner and outer surfaces of the ingot when the temperature of the inner wall of the ingot reached a maximum value of 744 °C.

**Figure 14:**
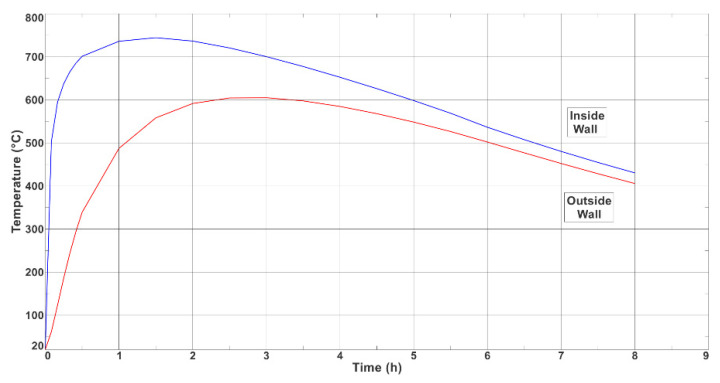
Distribution of temperature fields at maximum heating point of inner wall of ingot mould.

Figure 14 shows the temperature-variation curves of the inner and outer parts of the longer wall of the ingot (halfway up).

The maximum temperatures of the inner and outer walls of the ingot were 744° and 605 °C, respectively; hence, an average temperature of 674.5 °C was assumed to be the maximum temperature that was reached by the samples during the cyclic heating–cooling treatment in the high-temperature furnace (the parameters of which are presented in Section 3.2).

### 4.3. Results of Ingot Mould Stress Calculations

**Figure 15:**
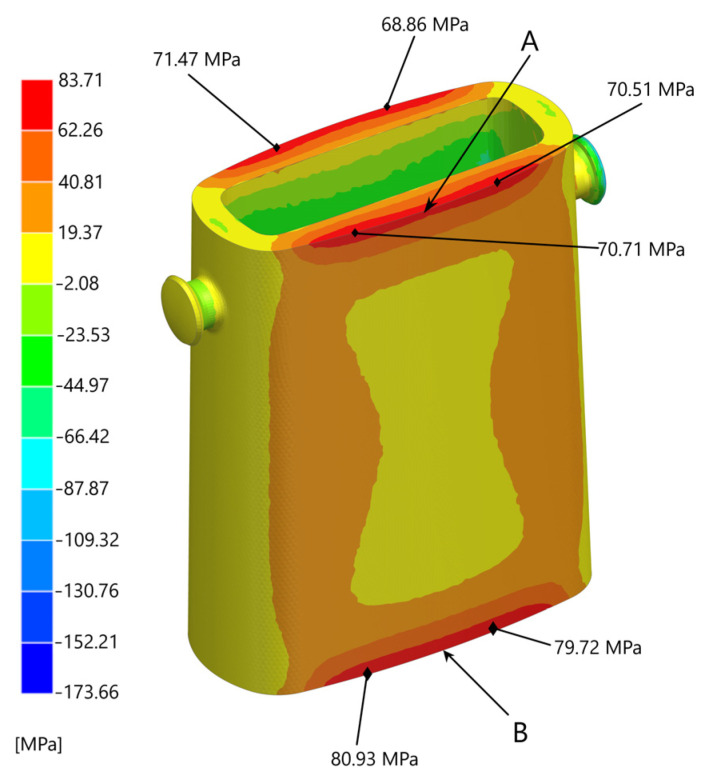
Stress field σ_x_ in walls of ingot after 7200 s from moment of metal filling.

The temperature changes that occurred during the ingot mould cycle caused changes in the deformation state and the stress in the walls of the ingot mould. The stress-state components were determined at time intervals of 300 s based on the stress model described in Section 3.3. The calculations were carried out using Simcenter NX with the Nastran module. The numerical determinations of the stress and strain components made it possible to identify any dangerous areas that were particularly prone to crack initiation and development. Figure 15 shows the stress field σ_x_ 2 h after filling the ingot with the alloy; this was after the central part of the outer wall of the ingot reached its maximum value during the production cycle.

**Figure 16:**
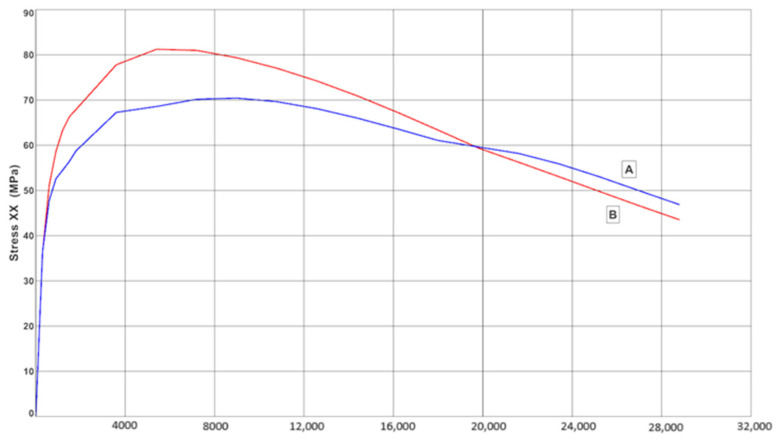
Changes in values of stress-state component σ_x_ at Points A and B marked in Figure 15.

Figure 16 shows the variation in the stress-state component σ_x_ that occurred at the edges of the ingot (the top edge is marked ‘A’ and the bottom edge ‘B’). These edges could result in areas of crack formation and development in the direction perpendicular to the stress vector σ_x_.

**Figure 17:**
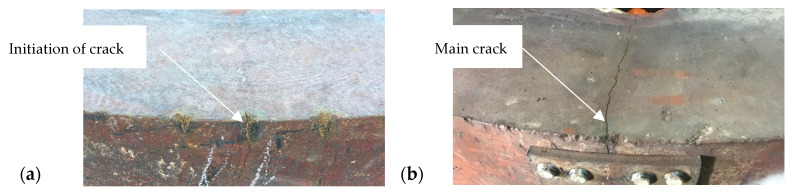
Fatigue fracture of ingot from steelmaking department: (**a**) crack initiation; (**b**) main crack.

This was particularly true of the bottom edge, where the stresses reached 81 MPa after 7200 s of pouring the alloy into the ingot. After this time, the stress values gradually decreased. Figure 17 shows an example of a resulting fatigue crack in an ingot mould that was used in a steelmaking department.

**Figure 18:**
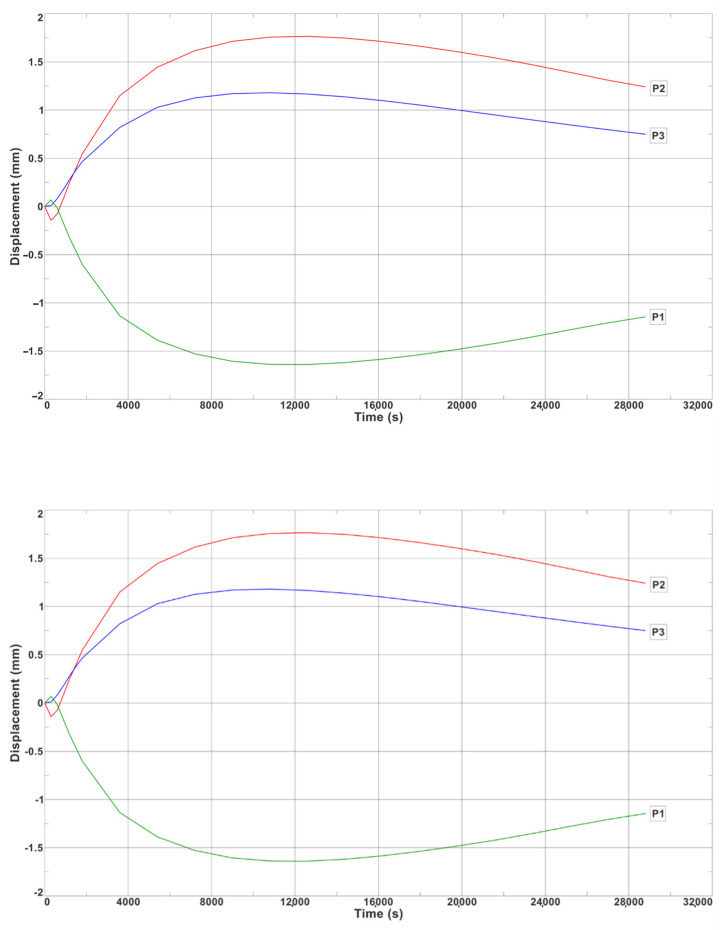
Changes in displacement of ingot wall determined numerically at Points P1, P2, and P3 (Figure 5b).

In turn, Figure 18 shows the numerically calculated displacement changes that occurred at Points P1, P2, and P3, which correspond to the displacement measurement locations on the actual bench (Figure 5b).

On the basis of the calculations that were carried out, it was found that the highest value of wall displacement (1.8 mm) occurred in the middle part of the ingot at Measurement Point P2 (Figure 18). This displacement caused the wall to deflect towards the centre of the ingot in the upper part of the ingot at Measurement Point P1 (Figure 18). The lower part of the ingot wall also deflected towards the centre of the ingot; at Point P3, the displacement was 1.3 mm. In contrast, the displacement of the upper part of the wall occurred in the opposite direction (i.e., outwards from the initial position before the flooding) and was −1.7 mm in length.

## 5. Results of Experimental Research

### 5.1. Result of Measurements on Pouring Test Bench

Prepared under the foundry’s production conditions, the pouring station for the actual ingot was identical to those that are usually used in steelmaking departments. Experimental tests were carried out on it to determine the temperature changes on the external surface of the ingot and the displacements of the longer side of the ingot during a production cycle. The temperature was recorded using a set of thermocouples, while the displacements were recorded using a specially made device. The research assumptions, including a description of the location of the temperature and displacement measuring points, are presented in Section 2.

#### 5.1.1. Measuring Temperature Changes

**Figure 19:**
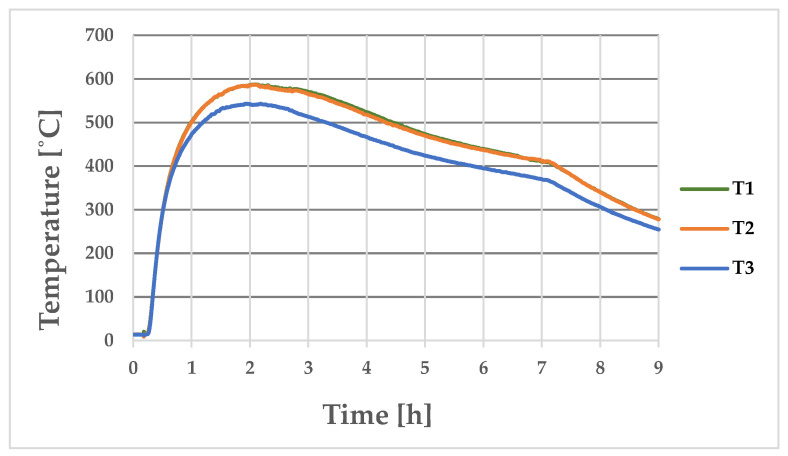
Temperature changes recorded with thermocouples on longer outer wall of ingot at Points T1, T2, and T3.

Figure 19 shows the temperature variations on the longer outer wall of the ingot that were recorded with the set of thermocouples at Points T1, T2, and T3.

**Figure 20:**
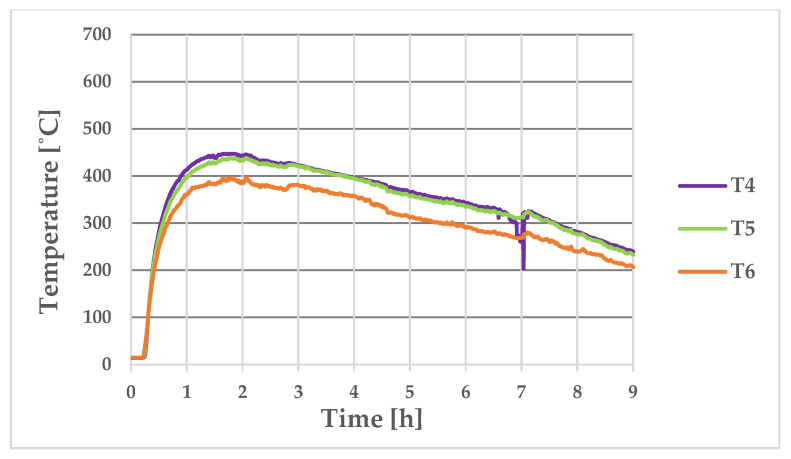
Temperature changes recorded with thermocouples on longer outer wall of ingot at Points T4, T5, and T6.

The maximum temperature on the outer wall of the ingot at Points T1 and T2 was 590 °C, while the lowest point (T3) reached 545 °C. The maximum temperature values occurred approximately 2 h after the flooding. Figure 20 shows the temperature changes at Points T4, T5, and T6 on the surface of the shorter wall of the ingot. As can be seen in Figure 20, an acceleration of the temperature drop can be observed 7 h after the pouring of the ingot cavity with the steel alloy. The time of 7 h was the moment when the steel ingot was knocked out and the free-cooling period of the ingot began.

The maximum temperature value that was recorded at Point T4 was 450 °C, while a slightly lower value of 446 °C was recorded at Point T5. Similarly to the temperature measurements on the longer side, the thermocouple that was located at the lowest point (T6) registered the lowest maximum temperature (399 °C).

**Figure 21:**
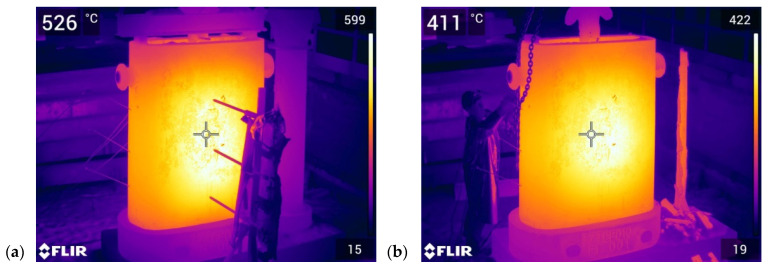
Inspection of ingot surface temperature made with FLIR T1020 thermal-imaging camera: (**a**) maximum ingot temperature during production cycle; (**b**) temperature immediately before ingot was knocked out.

The temperature measurements were controlled with an FLIR T1020 thermal-imaging camera that was positioned a short distance from the ingot; it took serial images every 5 min. Figure 21 shows the thermal images that were taken by the camera on the bench, Figure 21a shows the ingot when the maximum external surface temperature was reached, and Figure 21b shows the surface temperature of the ingot wall immediately before the steel ingot was knocked out. The temperatures that are shown in the top-left corner of each thermal image are the values that were recorded at the measurement marker location. In contrast, the values in the top-right corners of the images indicate the maximum recorded values in the entire image areas.

The maximum recorded ingot temperature was 599 °C; this occurred 2 h after the ingot was flooded. The maximum temperature that was recorded immediately before the ingot was knocked out (7 h after the flooding) was 422 °C.

#### 5.1.2. Displacement Measurement

**Figure 22:**
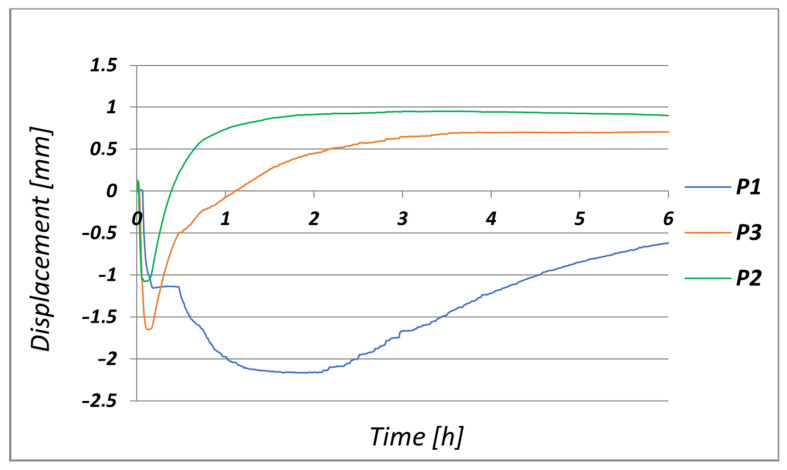
Displacements of ingot wall at Points P1, P2, and P3, recorded on test bench.

Figure 22 shows the changes in the displacements of the ingot wall at Points P1, P2, and P3 that were recorded with the inductive sensors during the production cycle.

### 5.2. Results of Cast Iron Strength-Reduction Measurements

**Figure 23:**
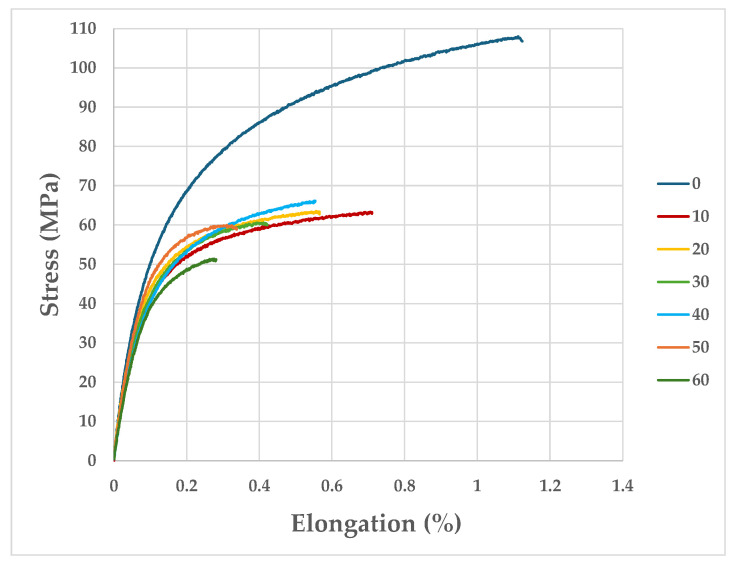
Typical curves that were obtained during static tensile testing of heat-treated ingot plastic specimens, along with reference specimen.

The tensile test procedure was carried out on cyclically heat-treated specimens. The specimens were heated to 675 °C for 60 min and then cooled to 20 °C for 320 min. The specimens were heat-loaded from 10 to 60 cycles. During the tensile test, the elongation and tensile force F were recorded. Figure 23 shows the typical strain–stress plots that were obtained during the static tensile test of the heat-treated ingot plastic specimens (within a range of 10 to 60 cycles), together with the reference specimen (0 cycles).

For the tested number of thermal load cycles (with intervals of 10 cycles), three tensile tests were carried out; the final result was the average of the tests that were carried out for the given range of the number of thermal load cycles. The differences in the obtained values of failure force F did not exceed ±0.3 kN in any of the tested cases. The fracture of each of the tested specimens occurred in the constriction zone of the specimen (the measuring zone).

As a result of the tensile tests, strain–stress function diagrams were obtained for each of the tested specimens. From these, the following values were determined: tensile strength (UTS), yield strength (R_p0.2_), Young’s modulus E, and Elongation A. These are presented as a function of the number of thermal load cycles.

Table 8 presents the obtained results for the basic mechanical parameters of the samples that were made from the ingot material subjected to thermal cycling within a range of 10–60 cycles, along with a reference sample (0 cycles).

**Figure 24:**
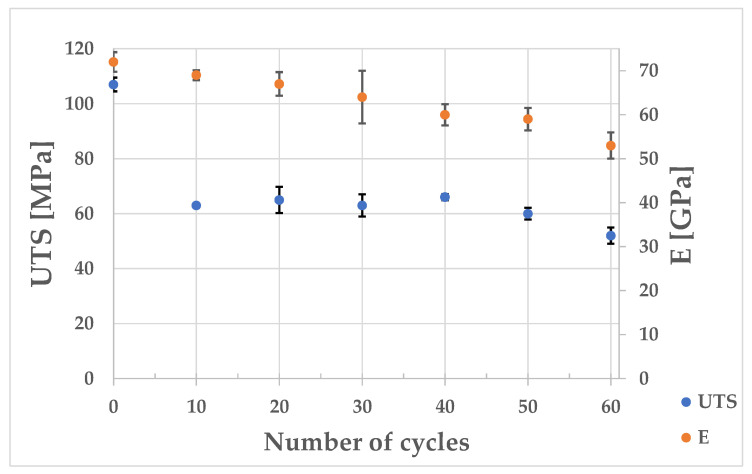
The dependence of UTS and Young’s modulus as functions of the number of thermal load cycles.

The tensile tests that were carried out showed that, after the first ten thermal loading cycles, there was a significant decrease in the tensile strength values (from 107 to 63 MPa). During the subsequent fatigue cycles, no decreases in the tensile strength values could be observed (between 10 and 40 cycles). When 50 and 60 loading cycles were reached, decreases of 6 and 14 MPa, respectively, in the tensile strength values could be observed compared to the strength of the sample that was subjected to 40 fatigue cycles. The dependence of UTS and Young’s modulus as functions of the number of thermal load cycles is shown in Figure 24.

### 5.3. Microstructure Test Results

**Figure 25:**
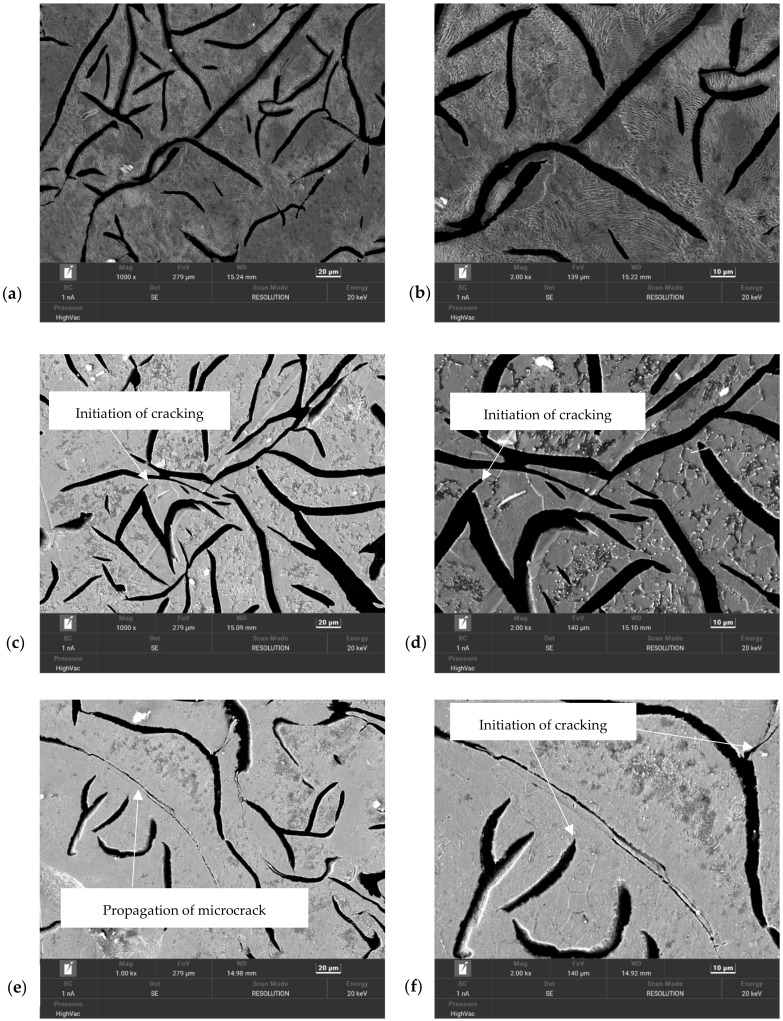

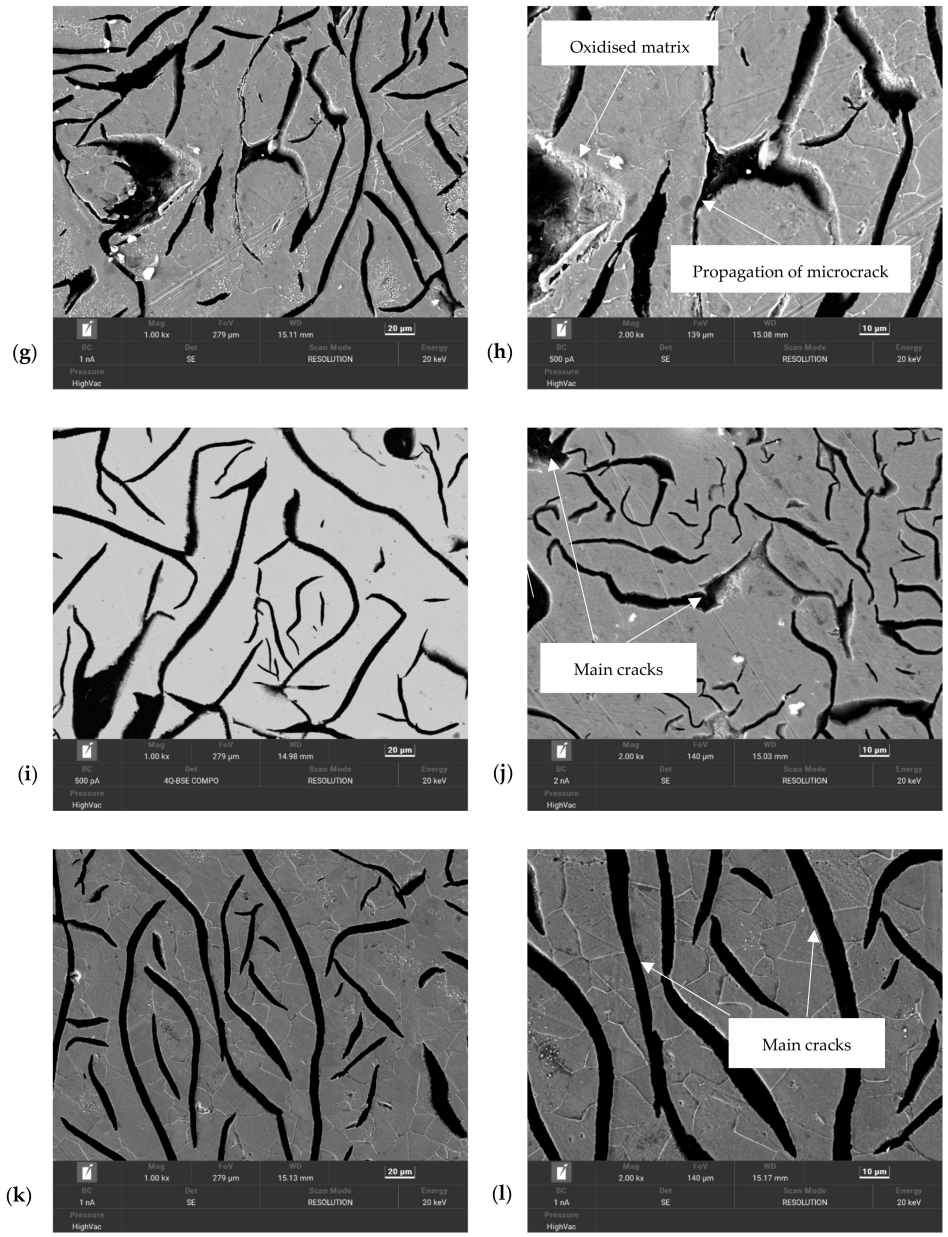

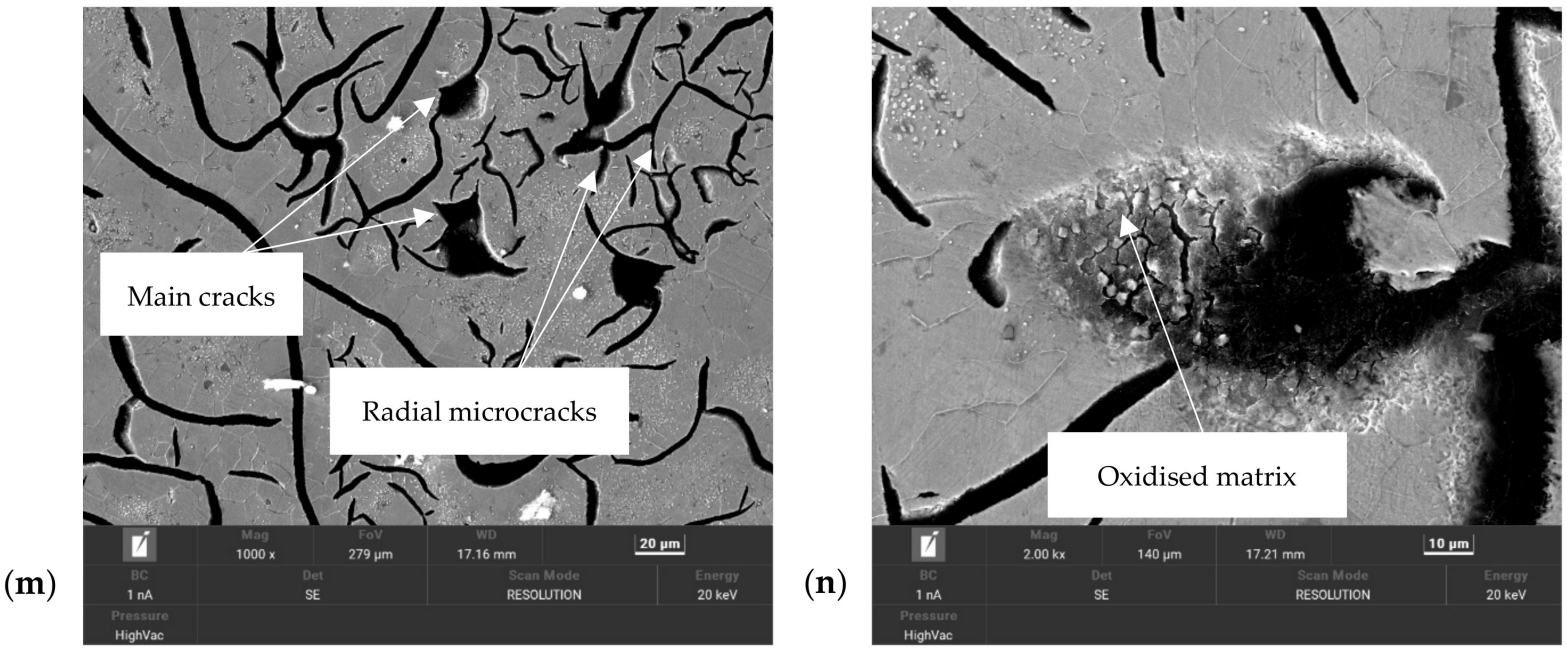
Electron microscopy images of grey cast iron microstructures of heat-treated samples: (**a**,**b**)—reference sample; (**c**,**d**)—10 cycles; (**e**,**f**)—20 cycles; (**g**,**h**)—30 cycles; (**i**,**j**)—40 cycles; (**k**,**l**)—50 cycles; (**m**,**n**)—60 cycles.

After the static tensile testing, the samples were prepared for microstructural analysis; the tests were performed via scanning electron microscopy (SEM) using a high-resolution Tescan Mira microscope (Brno, Czech Republic) with an FEG electron source (the beam energy was 20 keV, and the surface was observed in secondary electron mode (SE) and backscattered electron mode (BSE) by an Oxford Instruments SDD Ultim Max EDS detector (Abingdon, UK)). The studies were carried out at magnifications of 1000 and 2000×. Figure 24 shows a summary of the surface images of the heat-treated heating–cooling specimens over a range of 10–60 cycles (along with the reference sample). The magnifications of the sample microstructure images were chosen so that the shapes of the graphite precipitates could be observed (Figure 25a,c,e,g,i,k,m). Figure 25b,d,f,h,j,l,n show the morphology of the specimen at an applied magnification of 2000×; at this level, the phenomenon of structural degradation under cyclic thermal loading can be observed.

Subjecting grey cast iron to cyclic heating–cooling heat treatment had a significant effect on strength loss, as well as on the formation and development of fatigue cracks. The first nuclei of the crack initiation could already be observed after 10 loading cycles (Figure 25c,d). After 20 cycles, further cracks were initiated; the propagation of the already-existing microcracks could also be observed (Figure 25e,f). In those specimens that were subjected to 30 loading cycles, propagating microcracks and the formation of oxide layers at the microcrack boundaries could be observed (Figure 25g,h). After 40 and 50 cycles, clear microcracks were already visible (Figure 25i,j,k,l). In the sample subject to 60 cycles, a clear network of radial microcracks could be observed, along with the separation of the oxide layers (Figure 25m,n).

## 6. Discussion of Results

In this paper, experimental studies and numerical calculations were carried out to analyse the phenomena that occur during the operation of an ingot mould that is designed for casting steel ingots. In order to effectively shape the strength of an ingot casting machine, it is necessary to know how its material properties degrade under varying thermal loads and to determine the values of the time-varying temperature field, displacements, deformations, and stresses that occur during its operation. This information can contribute to increased ingot life as a result of taking the casting design process into account. The increased durability of an ingot casting can be achieved, among other approaches, by eliminating or significantly reducing the extreme stresses that are caused by the uneven heating of the walls; the inhibition of thermal expansion by lowering the maximum temperature of the ingot walls so as to minimise the proportion of permanent deformations; and by meeting the condition of the homogeneity of the stress–strain field on the external surface of the ingot walls.

Experimental tests were carried out on an actual pouring station in order to determine the temperature changes in the walls of an ingot and measure the displacements of the walls during the steel filling, heating, and cooling processes. The results of these tests were used to build a numerical model of the ingot and verify the results of the temperature, displacement, and stress calculations.

Figure 19 shows the temperature-change curves that were recorded on the outer wall of the ingot using thermocouples at Points T1, T2, and T3, which were placed along the longer wall of the ingot. The maximum temperature value at T1 and T2 was 590 °C, while at T3, it was 545 °C. On the other hand, Figure 11 shows the results of the numerical calculations of the temperature changes at the analogous measurement points; the maximum temperatures were 605 °C, 622 °C, and 510 °C for Points T1, T2, and T3, respectively. The differences between the thermocouple readings and the results that were obtained in the numerical simulation were not great: 15 °C, 32 °C, and 35 °C for Points T1, T2, and T3, respectively.

Figure 20 shows the temperature variations that were recorded on the outer wall of the ingot using the thermocouples at Points T4, T5, and T6, which were located along the shorter wall of the ingot. The maximum temperature values at Points T4, T5, and T6 were 450 °C, 445 °C, and 399 °C, respectively. Figure 12 shows the results of the numerical calculations for the corresponding measurement points; the maximum temperatures that were calculated at Points T4, T5, and T6 were 505 °C, 475 °C, and 400 °C, respectively. The differences between the thermocouple readings and the results that were obtained in the numerical simulation were not great (55 °C, 30 °C, and 1 °C for Points T1, T2, and T3, respectively). The greatest difference between the measurements and the numerical simulation results was 12% (occurring at Point T4).

The agreements between the experimental results and numerical calculations were confirmed by a control measurement that was made with a thermal-imaging camera. Two hours after flooding the ingot, the thermal-imaging camera at Point T2 indicated a temperature of 599 °C, while the thermocouple measurement gave a result of 590 °C and the FEM calculation was 622 °C. The measurement was repeated at 7 h after pouring (when the ingot was knocked out). In this case, the thermal-imaging measurement at T1 recorded a temperature of 422 °C, while the thermocouple measurement gave a result of 410 °C and the FEA calculation showed 450 °C.

Figure 14 shows the temperature-change curves on the surfaces of the inner and outer walls of the ingot at half the height of the longer side of the ingot. The inner wall of the ingot box heated up to 744 °C, while the outer wall heated up to 605 °C. The average temperature from the maximum heating values of the inner and outer walls was 674.5 °C. This value was taken as the maximum of the cyclic treatments of the ingot cast iron samples that were carried out.

Figure 22 shows the experimentally determined displacement changes of Measurement Points P1, P2, and P3. The greatest displacement was recorded at Point P1 (2.2 mm). At Points P2 and P3, the displacements were 0.95 mm and 0.73 mm, respectively. The return of the displacement vector of P1 was opposite to those of Points P2 and P3. This arrangement of the displacement of the measuring points caused the middle and lower parts of the ingot wall to bend inwards towards the heat source (which was the cooling ingot). On the other hand, the upper part of the ingot bent outwards in relation to its initial position before the flooding. Figure 18 shows the displacements that were determined by the numerical calculations for those points with the same coordinates as the measured points. As with the experimental measurements, the greatest displacement occurred at Point P2 (1.75 mm). For Points P1 and P3, the displacement values were 1.7 and 1.35 mm, respectively. The differences between the experimental measurements and the FEM-determined displacement values were 0.5, 0.8, and 0.62 mm at Points P1, P2, and P3, respectively. The correctness of the numerical calculations was confirmed by the correspondence between the experimentally and numerically determined displacement functions at each of the analysed points.

The temperature changes that occurred during the ingot’s mould cycle generated instantaneous changes in the strain–stress field in the walls of the ingot mould. Based on the analysis of the stress state of the walls of the ingot, the locations of those areas where cracks were initiated and developed were determined. Figure 15 shows the values of the component of the stress state σ_xx_ that occurred in the walls of the ingot after a period of 2 h after it was flooded with the melt. The edges of the connections between the outer wall and the top and bottom planes were dangerous places and were particularly prone to crack formations and development. The highest instantaneous stress values of 82 MPa occurred in the vicinity of the bottom edge 2 h after pouring the alloy into the ingot. In the vicinity of the upper edge, the stress values were slightly lower (around 72 MPa). Of all of the components of the stress tensor, only σ_xx_ was analysed (as this was the stress that caused the stretching of the ingot mould walls). Therefore, it caused crack propagation to develop perpendicularly to the direction of this stress. The analysed stresses arose from the inhibition of the thermal expansion of the ingot mould walls and should be referred to as mechanical stresses that were induced by temperature changes. On the other hand, the cyclic temperature changes of the inner surfaces of the ingot walls induced volume changes in some parts of the ingot; these were associated with the changes in the microstructure. In spite of the compressive stresses that occurred at the contact surface of the ingot wall with the liquid metal, the cyclic strain and stress changes that occurred at the graphite–matrix separation boundary resulted in the formation of a crack network (as was determined by the macroscopic stress model). On the one hand, the resulting crack network increased the resistance of the cast iron to the thermal shock that occurred during the filling of the ingot; on the other hand, the development of the microcracks led to them combining with the cracks that propagated from the outer surface of the ingot, thus causing the entire wall to crack.

In order to study the structural changes and kinetics of the crack development in the inner zone of the ingot surface, scanning studies of the microstructure of the cast iron were performed after it was subjected to different numbers of thermal-loading cycles (analogously to those of the real object); these are shown in Figure 25. As a result of the observation of the microstructure, it was found that the cyclic temperature changes caused significant changes in the structure of the ingot cast iron. The first stage was the initiation of the microcracks that are visible in Figure 25c,d,f. During the second stage, oxide separations were formed, along with the propagation of microcracks (the initiation of which took place in the previous stage—Figure 25e,h). In the third stage, the macrocracks were already visible; they ran along the graphite and matrix separations (as shown in Figure 25j,l,m). In the fourth stage, there was a further significantly greater intensification of the phenomena that occurred in the previous stages. The above-described changes in the microstructure of the ingot cast iron under the influence of increasing numbers of thermal loading cycles were directly related to the decreases in the strength properties of the cast iron. As shown in this study (based on an analysis of the curves that were obtained during the static tensile test shown in Figure 23), the strength properties of the ingot material decreased from 107 to 63 MPa after 10 cycles. The subsequent thermal fatigue cycles ceased to cause such pronounced drops in the strength. After another 40 cycles, the drop in the strength was only 3 MPa. The next 10 cycles caused a much greater decline (52 MPa). The strength tests (Table 8) showed that, after 60 floods, the tensile strength of the ingot material decreased by 52%, the yield strength by 33%, and the elastic modulus by 27% (shown in Figure 24). These changes should be taken into account, both in terms of the numerical model of thermal loads and in the strength-shaping of the ingot casting.

According to Hasselman [43], the thermal fatigue resistance of cast iron is proportional to tensile strength (UTS) and inversely proportional to the elastic modulus E determined at an ambient temperature. The authors of [4] also confirmed a similar relationship in their study. In the case of grey cast iron with a varying matrix, tensile strength had the greatest influence on the changes in the thermomechanical parameters [27,44]. The thermal fatigue resistance criteria that were cited in the above-mentioned publications can only be used to compare the fatigue resistance of different cast iron grades; they do not determine any changes in the UTS strength nor elastic modulus E under the action of the cyclic temperature changes that occur in ingot moulds. The research that was undertaken by the authors in the present study was aimed at demonstrating the relationship between number of thermal fatigue cycles and the changes that occurred in the microstructure of the cast iron from which the ingot was made (as well as its mechanical properties).

In industrial practice, the design of new ingot mould types is usually based on similar existing and proven solutions; any anomalies that occur during operation are corrected by making appropriate adjustments to the prototypes. This kind of trial-and-error method is costly and time-consuming; hence, some empirical relationships have been developed to facilitate the selection of certain design parameters, such as the ratio of ingot mould weight to ingot weight, wall thicknesses, and the radii of the ingot’s corner roundings. However, the high inaccuracy of the results that are obtained from these dependencies does not allow for the precise determination of the dimensions and shape of ingot mould structures. The biggest drawback of the above method is the omission of the analyses of the physical, mechanical, and chemical phenomena that occur during the cyclic heating and cooling of ingot mould casting. The studies that were carried out in this paper take the influence of material and design factors into account when determining the durability of the ingot moulds; hence, their results should contribute to the optimisation of the design of ingot moulds and, thus, to an increase in their durability and a reduction in their production costs. They should also result in decreases in harmful emissions due to reducing the number of defective ingot moulds.

The methodology that was presented in this paper is intended to be a tool for developing a method for predicting the fatigue life of ingot moulds. The fatigue life of a zone that is subjected to strong heating can be determined on the basis of the tests described in this paper; these were aimed at determining decreases in the strength of materials under the influence of increasing numbers of temperature cycles. This decrease in strength is related to the changes in the volume of the components of the structure at a temperature of about 700 °C, as well as to the oxidation process; these also affect the increase in volume of cast iron. Oxygen mainly penetrates through the gaps that are formed between the graphite and the matrix when volume changes in individual structural components occur, thus forming oxides (Figure 25h). Changing the form of the graphite is one of the factors that can help reduce the intensity of the oxidation process; also, reducing the proportion of the elements whose oxidations are causes of large increases in the volume of the cast iron can contribute to increases in the resistance of the material to thermal fatigue. In many cases, however, the changes in the chemical composition and form of the graphite precipitate structure (which result in a reduction in the intensity of the oxidation process) cause unfavourable changes in other thermal and mechanical properties of the ingot material or have an impact on increasing the cost of its manufacturing. In turn, an analysis of the stress state of the external surfaces of an ingot mould’s walls can be used to determine their shape and thickness so as to reduce stress values and eliminate any local extremes.

## 7. Summary

This article presents the results of experimental tests and numerical calculations that were carried out to analyse the phenomena that occur during the operation of an ingot mould that is designed for the casting of steel ingots. By means of experimental and numerical studies, the changes in the temperature of the ingot and the displacements of its walls during the operating cycle were determined. Using these results, the FEM method was used to determine the fields of the displacements, deformations, and stresses in the walls of the ingot during the pouring and cooling of the steel ingot. The basic strength properties of the ingot material were determined as functions of the numbers of heating–cooling heat-load cycles, and the effect of cyclic temperature changes on the microstructure of the cast iron was studied.

After conducting the above research, the following conclusions were formulated:

- A very good correspondence was found between the results of the temperature and displacement measurements of the ingot walls that were carried out at the foundry’s experimental pouring station and the results of the FEM calculations.
- The convergence of these results is considered to be a factor that verifies the correctness of the FEM calculations of the values of the field components (displacements, strains, and stresses) of the walls of the ingot during the operating cycle.
- On the basis of the experimental studies, the influence of the cyclic thermal loading of the ingot on the reduction in the strength properties of the ingot material was found to be very strong.
- This influence was particularly marked in the initial period of the ingot’s operation: after 10 cycles of thermal loading, the strength decrease was as high as 40%.
- The reduction in the strength of the ingot’s material was influenced by the changes that occurred in the microstructure after being under the influence of increasing numbers of thermal loads.
- An analysis of the microstructure images showed that these changes could be divided into four consecutive stages: the initiation of microcracks, the formation of oxide precipitates and the propagation of the microcracks (the initiation of which took place in the previous stage), the merging of the microcracks into macrocracks that ran along the graphite precipitates, and the rapid intensification of the phenomena that occurred in the previous stages.
- On the basis of an experimentally verified numerical model, it was possible to identify any dangerous places that were particularly prone to crack formation and propagation and, considering a broader perspective, to numerically optimise the structure of the ingot in order to improve its performance.
- When analysing the fatigue life of an ingot, it is necessary to take the influence of all of the components of the stress-state tensor into account—especially those that can affect crack propagation.
- The influence of any mechanical stresses that are caused by the inhibition of the thermal expansion of the external walls of an ingot (the value of which can be determined by the FEM method) and the thermal deformation of the internal walls that are in direct contact with liquid metal should be analysed separately.
- Thermal fatigue analyses of the inner walls of an ingot that are carried out on the basis of models of this phenomenon should take the changes that occur in the microstructure of the ingot material into account.
- An analysis of the effect of cyclic thermal loading on the microstructure of ingot materials makes it possible to determine the locations of crack formations and the manner of their propagation, as well as to relate these phenomena to experimentally determined decreases in the mechanical properties of ingot material that is under the influence of cyclic changes in the temperature field.

## Figures and Tables

**Table 1:** Chemical composition of studied grey cast iron based on spectral analysis (wt.%).

C	Si	Mn	P	S	Fe
3.81	1.63	0.72	0.03	0.03	Bal.

**Table 2:** Thermal treatment parameters of samples in high-temperature furnace.

Cycle Phase	Starting Temperature (°C)	Ending Temperature (°C)	Increase/Decrease in Temperature (°C/min)	Time (min)
Heating	20	750	25	35
Stabilisation	750	750	0	5
Cooling	750	20	2.5	320

**Table 3:** Cycle stages adopted in thermal model.

Step	Name	Starting Point (s)	Time of Completion (s)
1	Filling ingot mould with alloy	0	3600
2	Solidification and cooling of alloy	3600	18,000
3	Formation of gap	18,000	21,600
4	Knocking out ingot	21,600	21,600

**Table 4:** Values of heat transfer coefficients that were used in the temperature model.

Step	Interaction Pair	HTC (W/m^2^·°C)	α (W/m^2^·°C)
1, 2	Ingot Mould/Steel Alloy	200	-
3	Ingot Mould/Steel Alloy	100	-
1, 2, 3, 4	Ingot Mould/Environment	-	15
1, 2, 3, 4	Steel Alloy/Environment	-	10
1, 2, 3, 4	Ingot Mould/Intermediate Plate	-	50
1, 2, 3	Steel Alloy/Intermediate Plate	-	50

**Table 5:** Thermophysical properties of grey cast iron and steel alloy.

Material	Conductivity		Density		Specific Heat	
Grey Cast Iron ^1^	T (°C)	λ (W/m·°C)	T (°C)	ρ (kg/m^3^)	T (°C)	c (J/kg·°C)
	20	54	7000		30	467
	500	48.5			500	741
	1160	40			725	1100
	1173	38			1160	844
	2000	38			1600	871
Steel Alloy ^1^	20	50	7829		20	448
	50	35			400	483
	700	29			800	521
	1600	27			1600	596

^1^ MagmaSoft software database Version 5.5.

**Table 6:** Thermophysical properties of air.

Material	Conductivity	Density	Specific Heat
Air ^1^	λ (W/m·°C)	ρ (kg/m^3^)	C_p_ (J/kg·°C)
	0.0263	1.2	1007

^1^ Simcenter NX software database.

**Table 7:** Mechanical properties that described constitutive model of grey cast iron.

Temperature	20 °C	400 °C	750 °C	1000 °C	1160 °C	2000 °C
R_m_ (MPa) ^1^	105	61	19	11	3	1
Young’s modulus E (Mpa) ^1^	74,000	55,000	22,000	19,000	18,750	2000
Poisson’s ratio ^1^	0.26	0.26	0.26	0.26	0.26	0.26
Linear expansion coefficient × 10^−6^ (1/°C) ^1^	12.12	11.91	11.91	11.17	−25.42	34.84

^1^ MagmaSoft interface database Version 5.5.

**Table 8:** Summary of obtained results of static tensile test.

Number of Cycles	UTS [MPa]	R_p0.2_ [MPa]	E [GPa]	A [%]
0	107	74	72	1.07
10	63	56	69	0.71
20	65	58	67	0.55
30	63	58	64	0.43
40	66	58	60	0.55
50	60	59	59	0.32
60	52	50	53	0.28

## Data Availability

The original contributions presented in the study are included in the article, further inquiries can be directed to the corresponding author.
